# Dendritic Cell-Like Cells Accumulate in Regenerating Murine Skeletal Muscle after Injury and Boost Adaptive Immune Responses Only upon a Microbial Challenge

**DOI:** 10.1371/journal.pone.0155870

**Published:** 2016-05-19

**Authors:** Florian Wirsdörfer, Jörg M. Bangen, Eva Pastille, Daniel Schmitz, Sascha Flohé, Beatrix Schumak, Stefanie B. Flohé

**Affiliations:** 1 Surgical Research, Department of Orthopedics and Trauma Surgery, University Hospital Essen, University of Duisburg-Essen, Essen, Germany; 2 Institute of Cell Biology (Cancer Research), Medical Faculty, University of Duisburg-Essen, Essen, Germany; 3 Institute of Medical Microbiology, University Hospital Essen, University of Duisburg-Essen, Essen, Germany; 4 Institute of Molecular Medicine, Immunology, and Parasitology (IMMIP), University of Bonn, Bonn, Germany; Oklahoma Medical Research Foundation, UNITED STATES

## Abstract

Skeletal muscle injury causes a local sterile inflammatory response. In parallel, a state of immunosuppression develops distal to the site of tissue damage. Granulocytes and monocytes that are rapidly recruited to the site of injury contribute to tissue regeneration. In this study we used a mouse model of traumatic skeletal muscle injury to investigate the previously unknown role of dendritic cells (DCs) that accumulate in injured tissue. We injected the model antigen ovalbumin (OVA) into the skeletal muscle of injured or sham-treated mice to address the ability of these DCs in antigen uptake, migration, and specific T cell activation in the draining popliteal lymph node (pLN). Immature DC-like cells appeared in the skeletal muscle by 4 days after injury and subsequently acquired a mature phenotype, as indicated by increased expression of the costimulatory molecules CD40 and CD86. After the injection of OVA into the muscle, OVA-loaded DCs migrated into the pLN. The migration of DC-like cells from the injured muscle was enhanced in the presence of the microbial stimulus lipopolysaccharide at the site of antigen uptake and triggered an increased OVA-specific T helper cell type 1 (Th1) response in the pLN. Naïve OVA-loaded DCs were superior in Th1-like priming in the pLN when adoptively transferred into the skeletal muscle of injured mice, a finding indicating the relevance of the microenvironment in the regenerating skeletal muscle for increased Th1-like priming. These findings suggest that DC-like cells that accumulate in the regenerating muscle initiate a protective immune response upon microbial challenge and thereby overcome injury-induced immunosuppression.

## Introduction

Necrotic cell death induced by tissue destruction triggers a “sterile” inflammatory response that is similar to the response to infection in terms of leukocyte infiltration and formation of pro-inflammatory mediators at the site of injury (reviewed in [1]). Reports on skeletal muscle damage induced by toxin or freeze injury have described the infiltration of granulocytes, monocytes/macrophages, dendritic cells (DCs), and myogenic cells into the injured tissue [2–4]. Whereas granulocytes and monocytes are considered to remove cellular debris and to support the restoration of intact tissue organization, the role of DCs in the regenerating muscle is less clear.

DCs are professional antigen-presenting cells (APCs) and are found in lymphoid and non-lymphoid tissues under steady-state conditions [5]. DCs are regarded as the sentinels of the immune system. Upon uptake of foreign antigens in the periphery, DCs migrate into the draining lymphoid organ, where they efficiently trigger antigen-specific T cell responses. Sensing of microbial agents through Toll-like receptors (TLRs) induces a process termed maturation of DCs, which is associated with the upregulation of costimulatory molecules, such as CD40 and CD86, and with the secretion of cytokines. The number of DCs, their state of maturation, and the microenvironment during antigen uptake are decisive for the degree of subsequent T helper (Th) cell priming in the lymphoid organ [6]. Increasing evidence suggests that immigrating antigen-loaded DCs may interact with resident DCs or with recruited natural killer (NK) cells in the lymph node to promote Th cell priming [7,8]. Activated Th cells increase the expression of CD25 and CD69, proliferate, and differentiate toward interferon (IFN) γ–secreting Th type 1 (Th1) cells; toward Th2 cells that release interleukin (IL) 4, IL-5, and IL-13; toward Th17 cells; or toward regulatory T cells [9].

We have established a clinically relevant murine model of mechanical contusion injury to the skeletal muscle. This model mimics the traumatic muscle injury of severely injured patients and does not require the application of any toxin that might affect cells of the immune system [10]. After mechanical injury to the gastrocnemius muscle, the concentration of skeletal muscle cell–specific molecules, such as myoglobin and creatine kinase, are rapidly released by dying cells and circulate in the blood [10]. Later on, characteristic signs of regenerating muscle such as expression of myogenin and centrally located nuclei in the muscle fibers are visible [11]. Using this model, we recently showed that skeletal muscle injury interferes with Th1 priming to antigens applied distal to the site of injury, a finding indicating that there exist immune-mediated processes beyond local reparation and regeneration [12]. Extending our previous work, we have now investigated the inflammatory process in injured skeletal muscle tissue during regeneration and have examined whether DCs that appear in the damaged muscle tissue possess migratory and Th cell-stimulatory properties. For the first time, we provide evidence that DCs that accumulate in regenerating skeletal muscle remain there “on demand,” but in the case of an infectious insult they migrate into the draining lymph node, where they boost an antigen-specific Th cell response and thereby overcome injury-induced immunosuppression.

## Materials and Methods

### Mice

Male 10- to 12-weeks-old BALB/c mice were obtained from Harlan Winkelmann (Borchen, Germany). Male 10- to 12-weeks-old TLR4^-/-^ mice (BALB/c background; breeding pairs were kindly provided by B. Ryffel, University of Orleans) and IFN-γ^-/-^ mice (BALB/c background) were bred at the local animal facility. Male 6- to 10-week-old DO11.10 mice that carry a transgene for the αβ T cell receptor for the amino acid (aa) sequence 323 to 339 of ovalbumin (OVA) were bred at the local animal facility of the University Hospital Essen. The mice were kept under specific pathogen–free conditions and had access to standard rodent food and water *ad libitum*. In total, 226 mice were used in this study. All animal research protocols were approved by the local animal ethics committee Landesamt für Natur, Umwelt und Verbraucherschutz (LANUV), North-Rhine-Westphalia.

### Mechanical muscle injury and antigen application

Mice were anaesthetized by intramuscular (i.m.) injection of 58 mg/kg body weight ketamine and 7 mg/kg body weight xylazine (both Ceva Santé Animale, Duesseldorf, Germany) into one forelimb. The mechanical muscle injury was induced as previously described [10]. Briefly, a 20-g drop mass was dropped once from a height of 1.20 m onto a cuboid of 1 x 2 x 1cm (w x l x h) that was previously positioned on the posterior part of the gastrocnemius muscle. Injury was induced on both hindlimbs. Sham-treated animals were only anaesthetized. The mice did not receive any analgesics. During the first 24 h after injury the mice were monitored at 6 h intervals but did not shown any signs of pain or distress. Initial experiments verified that the induction of muscle injury neither damaged the lymphatic vessels nor induced bone fractures. The skeletal muscle damage led to a similar rise of troponin and creatine kinase levels in the serum indicating the reproducibility of the tissue damage in this injury model [10]. At different time points after sham treatment or injury the mice were sacrificed by cervical dislocation.

Depending on the experimental set up, mice received applications of unlabeled OVA protein (>100 endotoxin units per mg protein; Sigma Aldrich, Deisenhofen, Germany), fluorescein isothiocyanate (FITC)-labeled OVA protein (40 endotoxin units per mg protein; Molecular Probes, Goettingen, Germany), Alexa Fluor 647-labeled OVA protein (30 endotoxin units per mg protein; Molecular Probes), or unlabeled endotoxin-free Endograde OVA protein (Hyglos GmbH, Bernried, Germany) either 24 hours (h), 4 days (d), or 7 d after injury or sham treatment. The content of lipopolysaccharide (LPS) in the distinct OVA preparations was quantified with the Limulus Amebocyte Lysate (LAL) QCL-1000 assay (Cambrex, East Rutherford, NJ, USA). We dissolved 30 μg of the respective OVA protein preparation in 60 μl phosphate-buffered saline (PBS) and injected it i.m. into each gastrocnemius muscle. To facilitate the distribution of the antigen in the muscle, we divided the OVA dose into three parts. In one experimental setting, 30 μg Alexa Fluor 647-labeled OVA was additionally supplemented with 5 μg LPS (*E*. *coli* 026:B6, Sigma-Aldrich) before it was injected into the gastrocnemius muscle. In some experiments, NK cells were depleted through intraperitoneal application of 50 μl anti–asialo ganglioside-monosialic acid (αGM-1) serum (Wako Chemicals, Neuss, Germany) 24 h before and 48 and 96 h after injury or sham treatment. Normal rabbit serum (received from the local animal facility) served as a control. In selected experimental settings, the mice received OVA-specific T cells from naïve DO11.10 mice (for preparation see below) 24 h before the application of OVA.

### Analyses of muscle tissue

Total gastrocnemius muscles were prepared, cut into small pieces, and digested with 4 μg/ml collagenase (Liberase Blendzyme 2, Roche Diagnostics GmbH, Mannheim, Germany) for 60 min at 37°C. The slurry was sequentially passed through a 40-μm and a 30-μm cell strainer. The cells were washed with PBS, and the pellet of one muscle was resuspended in 500 μl PBS. We used 100 μl for flow cytometry (see below).

### Culture medium

As culture medium we used very low endotoxin VLE-RPMI 1640 medium supplemented with 10% fetal calf serum (FCS), 10 mM HEPES, 2 mM glutamine (all from Biochrom, Berlin, Germany), 0.06 mg/ml penicillin, 0.02 mg/ml gentamicin, and 0.05 mM 2-mercaptoethanol (all from Sigma). For lymph node cells from mice that had received bone marrow–derived DCs (BMDCs), we replaced the FCS in the culture medium with 1% normal mouse serum (PAA Laboratories, Pasching, Austria) to prevent the restimulation of FCS-specific T cells.

### OVA-specific T cell activation *in vivo*

T cells from spleens of naive DO11.10 mice were purified with the Pan T Cell Isolation Kit (Miltenyi Biotec, Moenchengladbach, Germany) and magnetic-activated cell sorting (MACS) according to the manufacturer’s instructions (>95% purity as assessed by flow cytometry). T cells were labeled with 1.5 μM carboxyfluorescein succinimidyl ester (CFSE; Molecular Probes) for 12 min at 37°C. After incubation for 30 min in culture medium, the cells were washed, and 5×10^6^ CFSE-labeled T cells were injected intravenously (i.v.) into recipient mice. After 24 h, unlabeled OVA protein or 1×10^5^ OVA-loaded BMDCs (see below) were administered i.m., and the popliteal lymph nodes (pLNs) were prepared after 3 additional days. Single-cell suspensions of pLN were obtained by flushing the lymph nodes with culture medium via a 27-g needle and digesting the remaining tissue with Blendzyme 2 (Roche Diagnostics GmbH). Cells were washed in culture medium and restimulated with 0.1 or 1 μg/ml OVA peptide (pOVA; aa 323–339; AnaSpec, San Jose, CA, USA). All cultures were set up in triplicate at 2×10^5^ cells per well (96-well plates, 200 μl per well). After 3 d, the concentration of IFN-γ, IL-10, and IL-2 in the supernatants was determined by enzyme-linked immunosorbent assay (DuoSet ELISA; R&D Systems, Wiesbaden, Germany). The 50% dilution of CFSE on daughter cells during the proliferation of OVA-specific T cells was assessed by flow cytometry.

### Confocal laser scanning microscopy

The gastrocnemius muscle was frozen in pre-cooled isopentane, and embedded in Neg-50 (Richard-Allan Scientific, Michigan, USA). Sections of 7 μm were fixed in 96% ethanol and were air-dried. After blocking with 1% BSA/10% normal mouse IgG (BD Biosciences), the sections were incubated with anti-Gr-1 (clone RB6-8C5) followed by a FITC-labeled anti-rat-IgG2b secondary antibody (1:2000; Bethyl Laboratories, Montgomery, Texas). Unspecific binding sites were blocked with rat serum and monovalent anti-rat IgG Fab fragments (1:50; Jackson Immuno Research, West Grove, PA) Thereafter, the sections were incubated with an antibody against Laminin-2 (α-2-chain, clone 4H8-2, 1:400; Alexis Biochemicals, Lorrach, Germany) and a Cy3-labeled donkey anti rat IgG antibody (1:200; Jackson ImmunoResearch, West Grove, PA). Between each step, the sections were rinsed with PBS before they were finally embedded in DAPI-containing Vectashield (Vector Laboratories). Images were taken using the confocal laser scanning microscope Axiovert 100 M with a LSM 510 Laser-Scanning-Module.

### Generation, labeling, and tracking of BMDCs

BMDCs were generated by culturing bone marrow cells from naïve mice with 20 ng/ml recombinant granulocyte/macrophage colony-stimulating factor (rGM-CSF; R&D Systems) as previously described [13]. On day 7, non-adherent cells were harvested, labeled with CFSE (0.5 μM for 12 min at 37°C), and loaded with 100 μg/ml unlabeled OVA protein (Sigma Aldrich) for 2 h. After being washed with PBS, 1×10^5^ OVA-loaded BMDCs were injected into the gastrocnemius muscle. The pLN cells were isolated after 48 hours.

### Flow cytometry

We stained pLN cells with fluorochrome-labeled antibodies against the T cell receptor specific for pOVA (clone KJ1-26; Caltag Laboratories, Burlingame, CA, USA) in combination with anti-CD4 (clone RM4-5), anti-CD69 (clone H1.2F3), and anti-CD25 (clone PC61.5). In one selected experiment, lymph node cells were stained with an antibody against CD49b (clone DX5). For tracking of OVA-FITC or OVA-Alexa Fluor 647, pLN cells were stained with antibodies against CD11c (clone N418) and CD11b (clone M1/70). Digested muscle tissue was stained with antibodies against CD11c, CD11b, Gr-1 (clone RB6-8C5), MHC class II (I-A^d^, clone 2G9), CD40 (clone HM40-3), CD86 (clone GL1), CD45 (clone 30-F11), Ly6C (clone HK1.4), and CD49b (clone DX5) in various combinations of 3 antibodies as indicated. To exclude dead cells Fixable Viability Dye (FVD) eFluor® 780 (eBioscience, Frankfurt am Main Germany) was used according to the manufacturers recommendations. For all stainings, appropriate isotype antibodies served as negative controls. All antibodies were obtained from BD Biosciences (San Diego, CA, USA).

To determine the absolute number of leukocytes per muscle, we analyzed stained muscle-derived leukocytes with TruCount tubes (BD Biosciences), which allow the measurement of a defined volume during flow cytometry. Information about the number of leukocyte subsets in the defined volume, together with information about the percentage of the cell subset among total leukocytes in the muscle, was used to calculate the absolute cell number per muscle. Flow cytometry was performed with a fluorescence-activated cell sorting (FACS) flow cytometer (FACSCalibur; BD Biosciences) and CellQuest Pro software (BD Biosciences) or with LSRII and DIVA software (both BD Biosciences).

### Statistical analyses

Data are expressed as mean±SD or median (25^th^ to 75^th^ percentile, range). Statistical differences between sham-treated and injured groups or between groups given various treatments were determined with Student’s *t*-test, analysis of variance (ANOVA), or, for values that were not normally distributed, the nonparametric Kruskal-Wallis test followed by Dunn’s post hoc test. Statistical significance was set at the level of p<0.05. Prism 5.0 software (Graph Pad Software, San Diego, CA, USA) was used for statistical analyses and for preparation of graphs.

## Results

### Appearance and maturation of DC-like cells in the injured tissue

**Fig 1:**
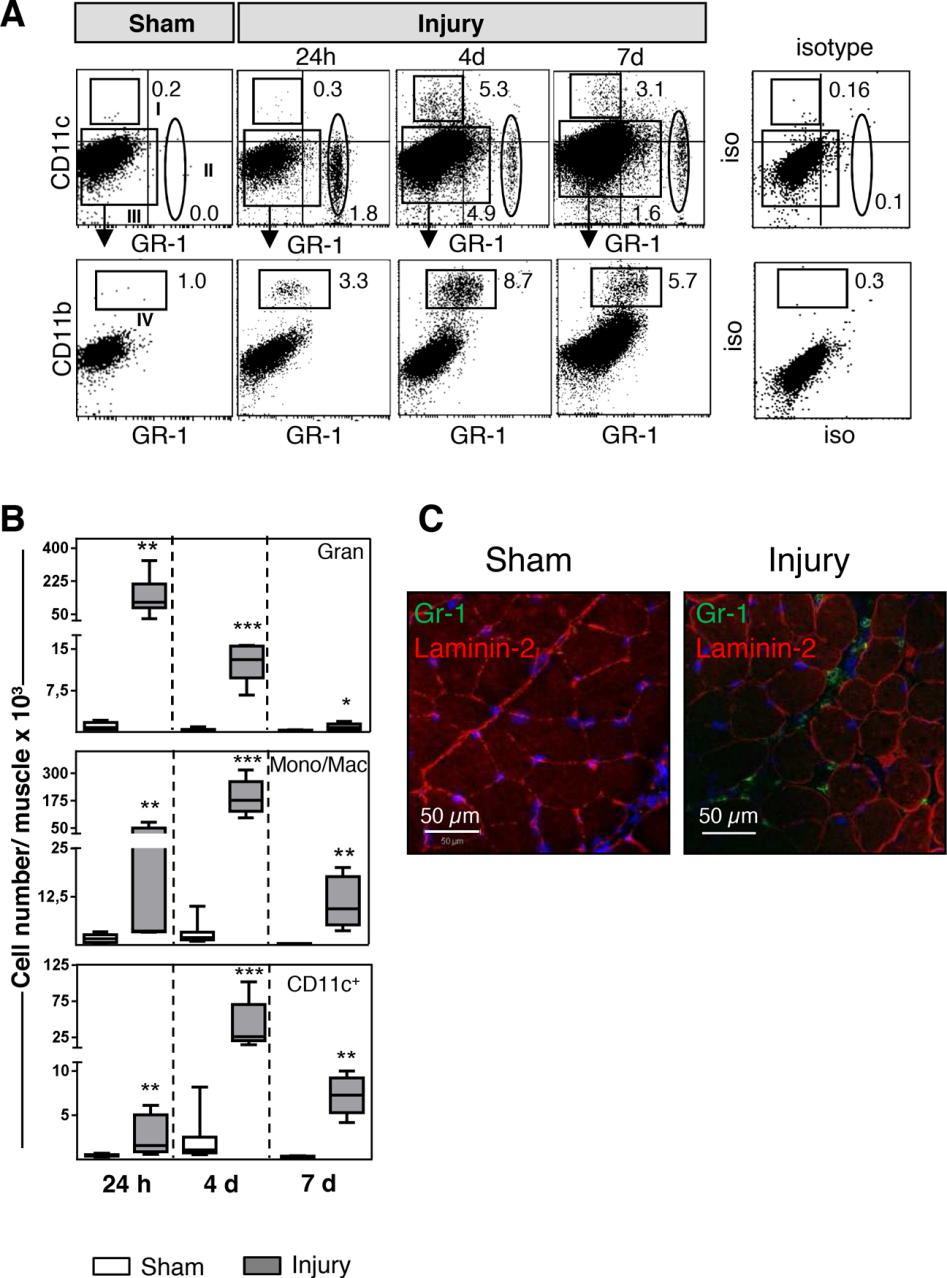
Infiltration of innate immune cells into the muscle after injury. **(A)** At indicated time points after injury or sham treatment, digested tissues of the gastrocnemius muscles were stained with fluorescent antibodies against Gr-1, CD11b, and CD11c. Region I depicts CD11c^+^Gr-1^–^ cells, and region II depicts Gr-1^+^CD11c^–^ neutrophilic granulocytes. Monocytes/macrophages (mono/mac) were identified as CD11b^+^ cells (region IV) among gated CD11c^–^Gr-1^–^ total muscle cells (region III). Numbers indicate the percentage of gated cells. The dot plots from sham-treated muscle are representative of dot plots from all sham-treated muscles at any time point. The dot plot for the isotype control is shown for time point 4d after injury and is representative for muscles from sham and injured mice at any time point. **(B)** The absolute number of granulocytes, monocytes/macrophages, and CD11c^+^ cells per muscle was determined. Median (horizontal lines), 25^th^ to 75^th^ percentile (extension of boxes), and range (error bars) of n = 6–8 mice per group are shown. Asterisks indicate statistically significant differences that were detected with the Kruskal-Wallis test followed by Dunn’s post hoc test. **(C)** Localization of granulocytes in the skeletal muscle 24 h after sham treatment or injury. Representative sections of skeletal muscle underwent immunofluorescent staining against Gr-1 (green) and laminin-2 (red) and were examined using laser scanning microscopy as described in Materials and Methods. Nuclei are visualized in blue. Scale bar = 50 μm **, p <0.01; ***, p <0.005 sham treatment vs. injury. iso, isotype.

First, we used flow cytometry to examine the leukocytes that infiltrated the skeletal muscle at various time points after mechanical injury (for the gating strategy, see Fig 1A). Sham-treated muscles contained nearly undetectable numbers of resident CD11c^-^Gr-1^+^ neutrophilic granulocytes, CD11c^-^CD11b^+^Gr-1^-^ monocytes/macrophages, and CD11c^+^Gr-1^-^ cells (Fig 1B). Within 24 h after injury, a large population of neutrophilic granulocytes had infiltrated the injured muscle, as had a small population of CD11c^+^ cells. The granulocytes were located in the large intercellular space that had formed after injury-induced breakdown of the tissue organization (Fig 1C). By 4 d after injury, monocytes/macrophages and additional CD11c^+^ cells had appeared in the muscle, whereas the population of granulocytes had declined. Monocytes/macrophages and CD11c^+^ cells were still present 7 d after injury, although to a minor degree, whereas granulocytes had nearly disappeared at that time point (Fig 1B).

**Fig 2:**
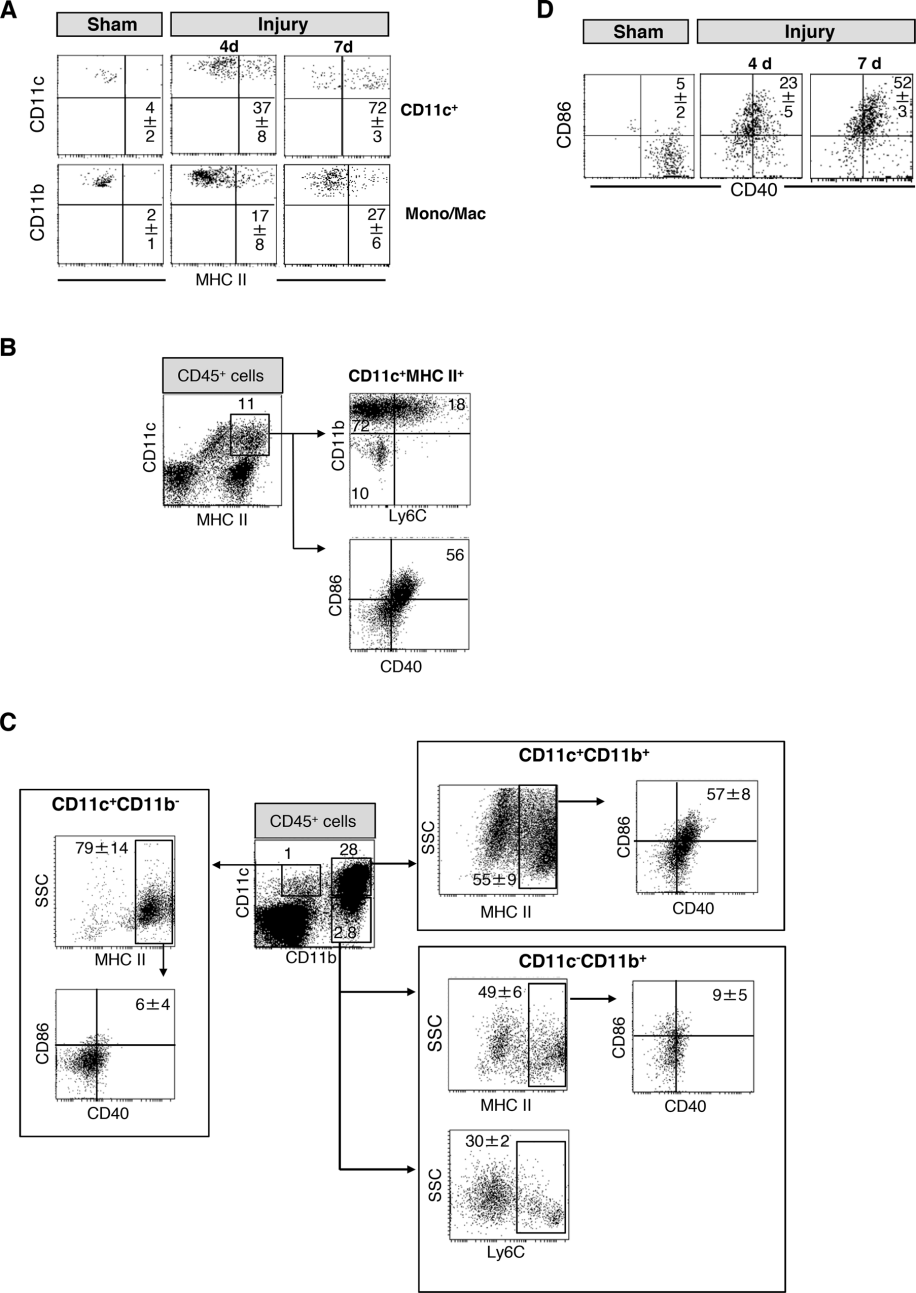
Phenotype of CD11c^+^ cells in the muscle after injury. **(A)** Four or 7 days after injury or sham treatment, cells from the gastrocnemius muscles were prepared, pooled per group, and stained for CD11c, CD11b, and MHC class II. Expression of MHC class II on gated CD11c^+^ cells and monocytes/macrophages (Mono/Mac). Representative dot plots are shown from n = 3 experiments each with n = 3 mice per group. **(B)** Expression of CD11b and Ly6C on gated CD11c^+^MHC class II^+^ cells 7 d after injury. The median (interquartile range) of the percentage of CD11c^+^MHC class II^+^ cells among total leukocytes was 11(8–15). **(C)** Expression of CD40 and CD86 on MHC class II^+^ cells among diverse subpopulations 7 d after injury. Among total leukocytes the percentage of CD11c^+^CD11b^+^, CD11c^+^CD11b^-^, and CD11c^-^CD11b^+^ cells was 14 (3–34), 0.9 (0.7–1.4), and 1.9 (1.2–2.9), respectively. Dot plots are representative for separate analyses of n = 6 muscles. **(D)** Pooled muscle cells were stained against CD11c, CD40, and CD86. The expression of CD40 and CD86 on gated total CD11c^+^ cells 4 and 7 d after injury or sham treatment was determined. Representative dot plots are shown from n = 3 experiments with n = 3 mice per group. Numbers in the dot plots indicate the mean±SD of the percentage of gated or double-positive cells.

We further investigated whether the population of CD11c^+^ cells in the injured muscle contained DCs. On day 4 after injury, 37% of the CD11c^+^ cells (approximately 7800 cells) expressed MHC class II and, thus, fulfilled the classic criterion of DCs (Fig 2A). On day 7 after injury, 72% of all CD11c^+^ cells were MHC class II^+^ [median (25–75% interquartile range) 7035 (5464–7379), n = 6]. In contrast, only a minor fraction of CD11c^-^ monocytes/macrophages expressed MHC class II molecules after injury (Fig 2A).

We analyzed additional markers on CD11c^+^ cells in the skeletal muscles 7 d after sham treatment or injury. CD45^+^ leukocytes were gated according to S1A Fig. Among CD11c^+^MHC class II^+^ cells, 90% also expressed CD11b (Fig 2B) and more than 50% resembled mature DCs according to their expression of the co-stimulatory molecules CD40 and CD86 (Fig 2B). A part of the CD11b^+^ DCs expressed the marker Ly6C (Fig 2B) pointing to a monocytic origin of these cells. The analysis of all Ly6C^+^ leukocytes identified a prominent population of CD11b^+^ cells that displayed an inhomogeneous expression of CD11c and MHC class II (S1B Fig). Almost all cells of the minor CD11c^+^CD11b^-^ population expressed MHC class II but did not express CD40 or CD86 (Fig 2C). One third of the CD11c^-^CD11b^+^ monocyte/macrophages expressed Ly6C (Fig 2C) but only few cells were positive for CD40 and CD86 (Fig 2C). On day 4 after injury, the percentage of CD40^+^CD86^+^ among total CD11c^+^ cells was much lower than on day 7 (Fig 2D). The skeletal muscles of sham mice contained only few leukocytes that were mainly CD11c^-^CD11b^+^. Some CD11c^+^ cell were detectable but this number was too low to allow the evaluation of the subset distribution as described above (see S1C Fig).

We also detected a very small population of NK cells in the injured skeletal muscle. These NK cells did not express CD11c (see S1D Fig). Thus, the CD11c^+^ cells that appear in the skeletal muscle after injury are composed of classical DCs and monocyte-derived cells.

To address the question whether TLR4 or IFN-γ played a role during injury-induced maturation of the DC-like cells in the injured muscle, the expression of CD40 and CD86 on CD11c^+^ cells from wildtype mice, TLR4^-/-^, and IFN-γ^-/-^ mice was analyzed 7 d after injury. The maturation of these cells in the muscle after injury depended neither on signaling through TLR4 nor on IFN-γ (see S2 Fig). Thus, CD11c^+^ cells accumulate in the skeletal muscle after mechanical injury and in part acquire the phenotype of mature DCs.

### Migration of DCs from the muscle into the pLN after microbial stimulation

**Fig 3:**
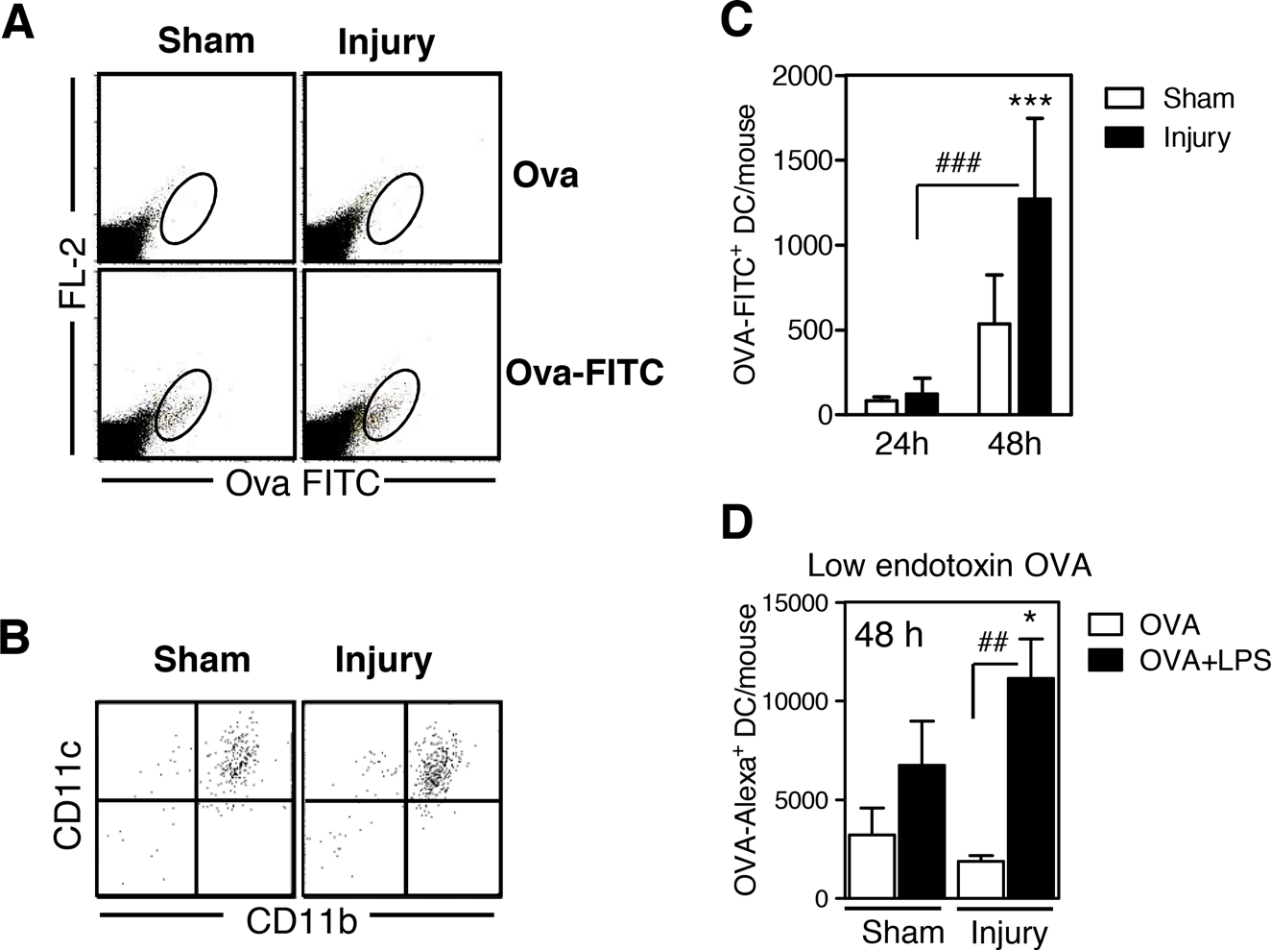
Dendritic cell-like cells in the injured muscle tissue take up ovalbumin after intramuscular application *in vivo* and migrate into the popliteal lymph node. Seven days after injury or sham treatment, unlabeled ovalbumin (OVA; Sigma), OVA-fluorescein isothiocyanate (FITC), or OVA-Alexa Fluor 647 was injected into the gastrocnemius muscles. After 24 or 48 h, total popliteal lymph node cells from individual mice were stained for CD11c and CD11b. The FL-2 channel remained free. **(A)** Representative dot plots of popliteal lymph node cells showing the gating strategy of OVA-FITC^+^ cells among total lymph node cells 48 h after OVA application. **(B)** CD11c and CD11b expression of gated OVA-FITC^+^ cells. **(C)** Absolute number of OVA-FITC^+^ dendritic cells (DCs) in the lymph nodes per mouse 24 h (n = 4 per group) and 48 h (n = 9 per group) after injury or sham treatment. **(D)** OVA-Alexa Fluor 647 alone or in combination with lipopolysaccharide (LPS) was injected, and the absolute number of CD11c^+^OVA-Alexa^+^ DCs in the lymph node was determined 48 h later (n = 4 per group). Data are presented as mean±SD. Symbols indicate statistical differences that were detected with analysis of variance (ANOVA). *, p<0.05; ***, p<0.001 sham treatment vs. injury. ##, p<0.01; ###, p<0.001.

Resident DCs in non-lymphoid tissue are known to take up antigens in the periphery and to transport them into the draining lymphoid organ for antigen-specific Th cell priming. To determine whether DC-like cells that had been recruited to the injured muscle behaved similarly, an LPS-containing preparation of FITC-labeled OVA was injected into the gastrocnemius muscle 7 d after injury or sham treatment. Unlabeled OVA served as a negative control. Forty-eight h after i.m. application of FITC-labeled OVA, we detected a population of FITC-positive cells in the pLN (Fig 3A). Virtually all of these FITC-positive cells in the pLN were CD11c^+^CD11b^+^ myeloid DCs (Fig 3B). At this time point, the pLN of injured mice contained 2.4-fold more OVA-FITC^+^ DCs than did the pLN of sham-injured mice (Fig 3C). In contrast, 24 h after OVA injection only a few OVA-FITC^+^ DCs were present in the muscles of both groups (Fig 3C).

To determine whether the migration of DC-like cells from the muscle into the lymph node was dependent on a microbial stimulus, we used OVA-Alexa-Fluor 647, which contains a lower LPS concentration than does OVA-FITC, for i.m. injection. In contrast to OVA-FITC, OVA-Alexa-Fluor 647 did not cause an increased migration of DCs into the pLN (Fig 3D). In contrast, supplementing OVA-Alexa Fluor 647 with LPS before i.m. application restored the increased migration of DC-like cells from the injured muscle into the pLN within 48 h (Fig 3D). Thus, upon challenge with a microbial stimulus, DCs transport foreign antigens from the injured skeletal muscle to the draining pLN.

### Increased Th1-like priming after injection of an antigen into the injured tissue

**Fig 4:**
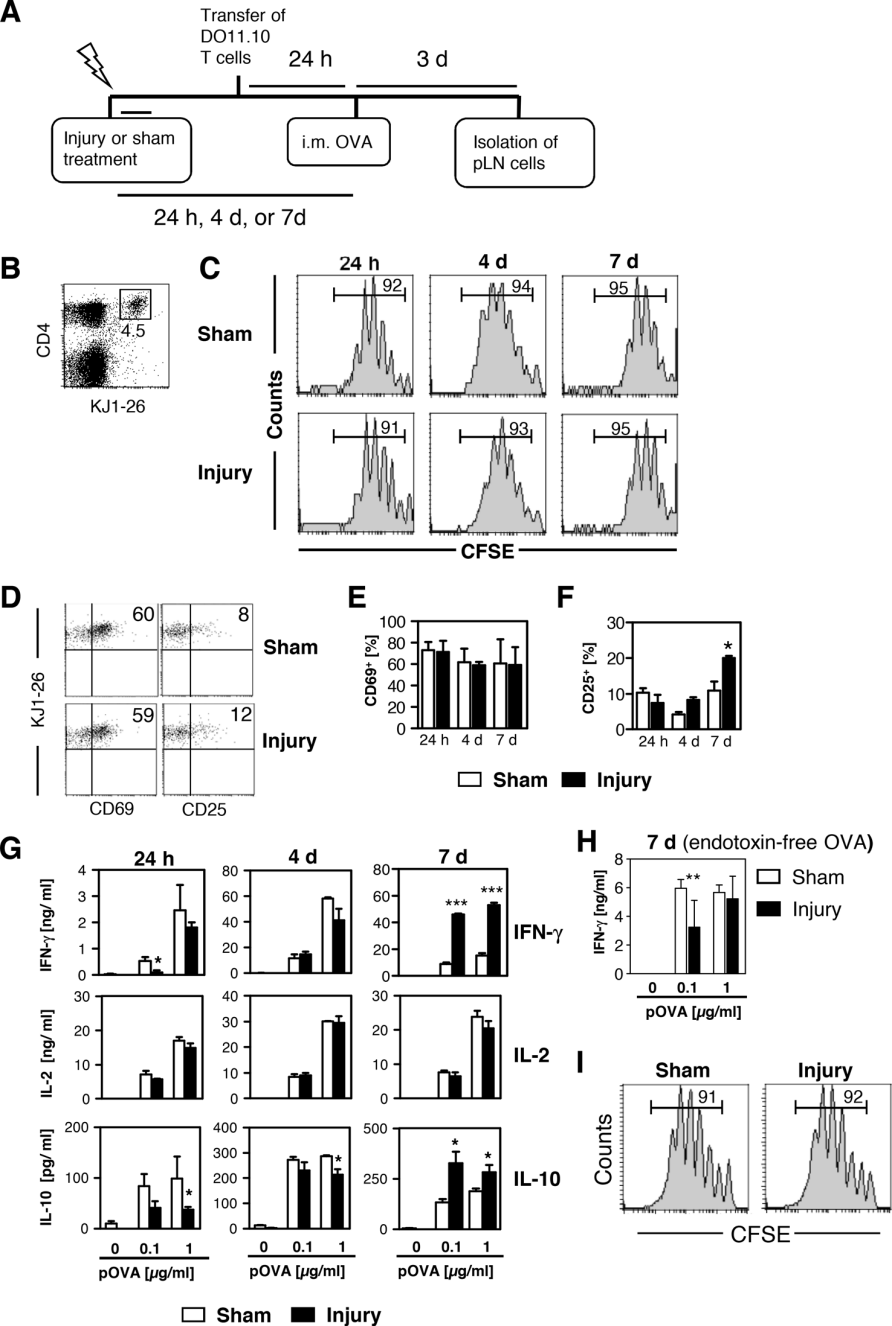
The injection of endotoxin-containing but not endotoxin-free OVA into the injured muscle increases T helper cells type 1-like priming in the popliteal lymph node. One, 4, or 7 days after injury or sham treatment, lipopolysaccharide (LPS)-containing unlabeled ovalbumin (OVA; Sigma) (A-G) or endotoxin-free OVA (Endograde) (H, I) was injected into the gastrocnemius muscles of mice that, 24 h earlier, had received OVA-specific T cells labeled with carboxyfluorescein succinimidyl ester (CFSE) from DO11.10 mice. Three days after OVA application, popliteal lymph node (pLN) cells were isolated and pooled by group. **(A)** Schematic overview of the experimental setup. **(B)** Gating of CD4^+^ OVA-specific (KJ1-26^+^) T helper (Th) cells among total lymph node cells. The number depicts the percentage of OVA-specific Th cells. **(C)** CFSE dilution in gated OVA-specific Th cells. Numbers indicate the percentage of cells that underwent at least one division. **(D)** Representative dot plots of the expression of CD69 and CD25 on gated OVA-specific Th cells. **(E)** Percentage of CD69^+^ and **(F)** CD25^+^ cells among gated OVA-specific Th cells on days 1, 4, and 7 after treatment. Data are presented as mean±SD of 3 experiments, each with n = 3 mice per group. **(G)** Lymph node cells were restimulated with OVA peptide (pOVA) *in vitro*, and the concentrations of interferon (IFN) γ, interleukin (IL) 2, and IL-10 in the supernatants were determined after 3 days. Data are presented as mean±SD of triplicate cultures and are representative of one of 3 experiments, each with n = 3 mice per group. **(H)** Content of IFN-γ in the supernatants from restimulated lymph node cells of mice that had received endotoxin-free OVA on day 7 after injury or sham treatment. Data are presented as mean±SD of triplicate cultures (n = 4 mice per group). **(I)** CFSE dilution in gated OVA-specific T helper cells among lymph node cells from mice that had received endotoxin-free OVA on day 7 after injury or sham treatment. Numbers indicate the percentage of cells that had undergone at least one division. Asterisks indicate statistically significant differences that were detected with two-way analysis of variance (ANOVA). i.m., intramuscular. *, p<0.05; **, p<0.01; ***, p<0.001 sham treatment vs. injury.

Considering the potential ability of DC-like cells in the injured muscle to act as APCs and to migrate into the draining pLN, we next investigated the antigen-specific Th cell response in the pLN after the injection of OVA into the gastrocnemius muscle. CFSE-labeled OVA-specific T cells were injected i.v. 24 h before a subsequent injection of unlabeled LPS-containing OVA into the gastrocnemius muscle. The injection of OVA was performed 24 h, 4 d, or 7 d after injury or sham treatment (for experimental design see Fig 4A). After 3 d, pLN cells were isolated, and OVA-specific Th cells were identified as CD4^+^KJ1-26^+^ cells (Fig 4B). At each time point of OVA injection into the muscles, OVA-specific Th cells in sham-treated and injured mice proliferated similarly (as shown by the reduction of the intracellular CFSE content; Fig 4C) and expressed CD69 to a comparable extent (Fig 4D and 4E). OVA-specific Th cells in injured mice expressed significantly higher levels of the activation marker CD25 than did Th cells in sham-treated mice when OVA had been injected on day 7 after the treatment (Fig 4F).

We next restimulated the pLN cells with pOVA *in vitro* and quantified the content of cytokines in the supernatants. In the absence of pOVA, pLN cells of both groups did not release significant levels of IFN-γ, IL-2, or IL-10 (Fig 4G). Restimulation with pOVA strongly induced the secretion of IFN-γ and IL-2 and to a lesser extent of IL-10 from pLN cells of either group (Fig 4G). Intracellular staining of IFN-γ revealed that the OVA-specific Th cells were the major source of IFN-γ in restimulated lymph node cells (see S3A Fig) in contrast to NK cells (see S3B Fig) that were present in the lymph nodes at less than 0.5% (see below). When OVA was applied 7 d after injury or sham treatment but not at earlier time points, pLN cells from injured mice secreted much higher amounts of the Th1-associated cytokine IFN-γ and also higher levels of IL-10 than did pLN cells from sham-treated mice (Fig 4G). The pLN cells of both groups released IL-2 equally at any time point (Fig 4G). None of the Th2-associated cytokines IL-4, IL-5, or IL-13 was detectable in the supernatants (see S4 Fig).

As shown above, the migration of DC-like cells from the injured muscle into the pLN was dependent on a microbial challenge (Fig 3D), and the increased Th1-like priming in the pLN was associated with the presence of mature DC-like cells in the injured muscle (Fig 4G). We next investigated whether the enhanced Th1-like priming in the pLN also occurred when a LPS-free preparation of unlabeled OVA was used for i.m. injection. Therefore, unlabeled LPS-free OVA was injected into the muscle 7 d after injury or sham treatment, and Th cell priming was analyzed as described in Fig 4G. pLN cells from injured mice did not show enhanced IFN-γ secretion after restimulation in contrast to pLN cells isolated after the administration of LPS-containing OVA (Fig 4H). The proliferation of OVA-specific Th cells in the pLN of both groups did not differ (Fig 4I). Thus, the presence of mature DC-like cells in the injured muscle and a microbial challenge at the time of OVA injection is associated with increased antigen-specific Th1-like priming in the draining pLN, whereas Th cell proliferation remains unaffected.

### The local microenvironment in the injured tissue prepares DCs for enhanced Th1-like priming

After OVA is injected into the muscle, a part of the soluble antigen may reach the pLN directly via the blood stream and may be presented by APCs that reside in the pLN in the steady-state, such as resident DCs. To address the relevance of circulating antigen, we applied OVA i.v. instead of i.m. 7 d after injury or sham injury. All animals had received CFSE-labeled OVA-specific T cells 24 h earlier. OVA-specific T cells only marginally proliferated in the pLN and in the spleen of both injured and sham-treated mice 3 d after OVA administration (see S5A Fig). However, neither IFN-γ nor IL-10 could be detected in the supernatants after restimulation of pLN or spleen cells (see S5B Fig).

**Fig 5:**
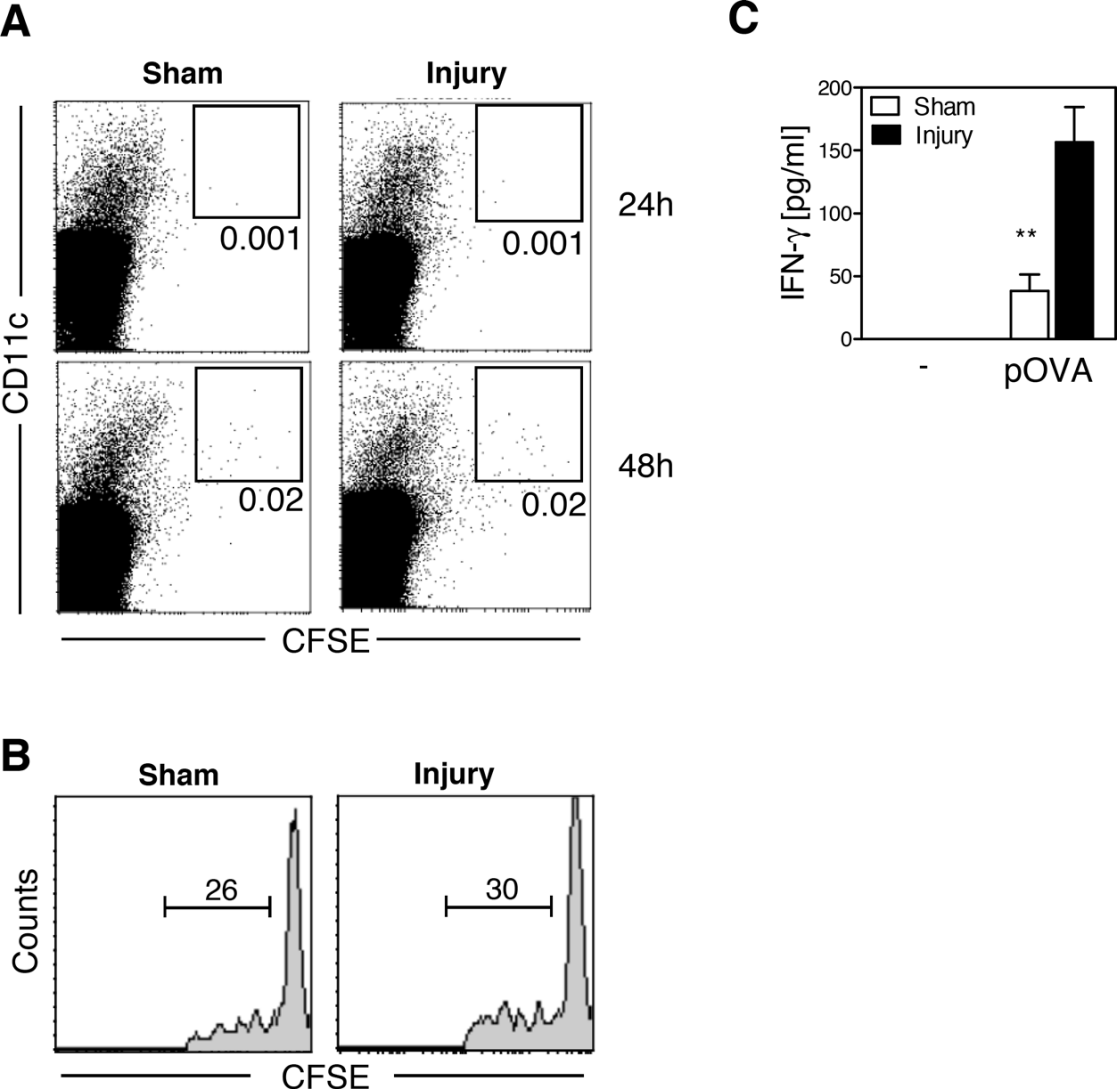
Dendritic cells exposed to the microenvironment in the injured muscle possess enhanced ability to prime T helper cells type 1 upon migration into the popliteal lymph node. Bone marrow-derived dendritic cells (BMDCs) labeled with carboxyfluorescein succinimidyl ester (CFSE) **(A)** or unlabeled **(B, C)** were loaded with lipopolysaccharide (LPS)-containing unlabeled ovalbumin (OVA) (Sigma) and were subsequently injected into the gastrocnemius muscles 4 days after injury or sham treatment. **(A)** Twenty-four or 48 h later, popliteal lymph node (pLN) cells were prepared and stained for CD11c. CFSE^+^ BMDCs were gated. Numbers indicate the percentage of CFSE^+^ BMDCs among total lymph node cells. **(B, C)** Before the injection of OVA-loaded BMDCs, the mice received CFSE-labeled OVA-specific T cells. After 3 d, pLN cells were isolated and pooled by group. **(B)** Representative histogram of CFSE dilution in gated OVA-specific CD4^+^KJ1-26^+^ T helper cells. **(C)** Lymph node cells were restimulated with 1 μg/ml OVA peptide (pOVA), and the concentration of interferon (IFN) γ in the supernatants was determined after 3 days. Data are presented as mean±SD of triplicate cultures and are representative of one of 3 experiments with n = 3 mice per group. Asterisks indicate statistically significant differences that were detected with Student’s *t*-test. **, p<0.01 sham treatment vs. injury.

Next, we investigated the impact of the microenvironment in the injured skeletal muscle on the T cell-stimulatory capacity of the DC-like cells that appeared at the site of tissue damage. *In vitro* with GM-CSF generated BMDCs are phenotypically equivalent to monocyte-derived DCs [14] that seemed to be present in the injured skeletal muscle (see S1B Fig). Therefore, BMDCs from naïve mice were labeled with CFSE, loaded with OVA, and injected into the gastrocnemius muscle of mice 4 d after injury or sham treatment, equivalent to the appearance of endogenous DC-like cells after injury (Fig 1). This approach further minimized the possibility that soluble OVA reached the pLN through the lymph vessels after i.m. injection and was taken up by resident DCs in the pLN. Between 24 h and 48 h after i.m. injection, BMDCs appeared to a similar extent in the pLN of both groups (Fig 5A) and induced a comparable proliferation of OVA-specific Th cells in the pLN of injured and sham-treated mice (Fig 5B). However, restimulated pLN cells from injured mice released higher amounts of IFN-γ than did cells from sham-treated mice (Fig 5C). Thus, the microenvironment in the injured muscle, the site of antigen encounter, seems to be decisive for the increased Th1-promoting ability of the DCs in the damaged tissue.

### NK cells are not involved in the increase of Th1-like priming after injury

**Fig 6:**
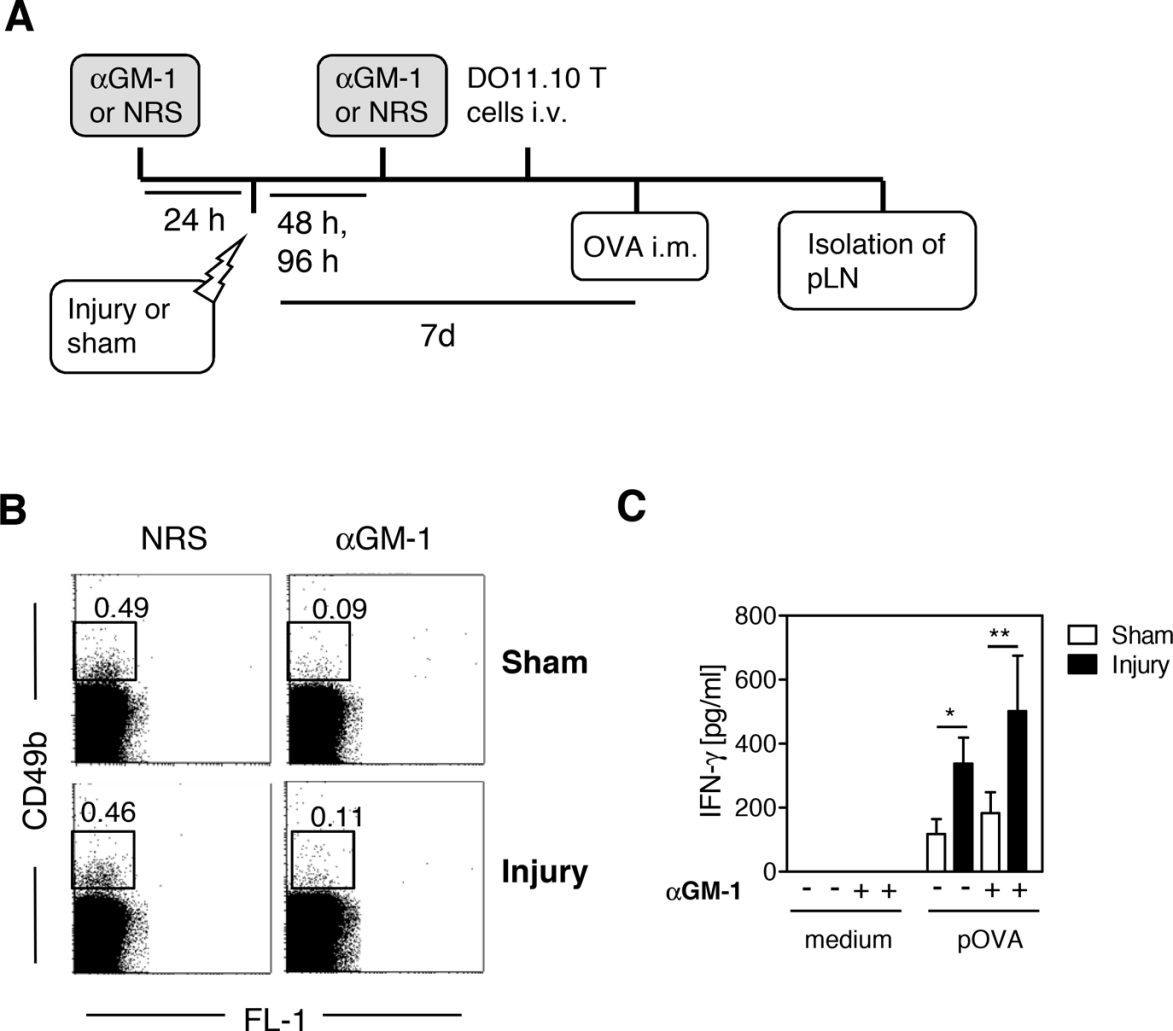
Natural killer cells do not contribute to enhanced T helper cell priming mediated by dendritic cells in the injured muscle. **(A)** Schematic overview on the experimental setup. Natural killer (NK) cells were depleted through intraperitoneal application of anti–asialo ganglioside-monosialic acid (αGM-1) serum 24 h before and 48 and 96 h after injury or sham treatment. Normal rabbit serum (NRS) served as a control. All mice received ovalbumin (OVA)-specific T cells from DO11.10 mice intravenously (i.v.) 1 day before OVA application. Unlabeled lipopolysaccharide-containing OVA (Sigma) was injected intramuscularly (i.m.) into the gastrocnemius muscles 7 days after injury or sham treatment. After 3 days, the popliteal lymph node (pLN) cells were pooled by group and were stained with antibodies against CD49b or were restimulated with OVA peptide (pOVA). The content of interferon (IFN) γ in the supernatants was determined 3 days later. **(B)** Representative dot plots with numbers indicating the percentage of CD49b^+^ NK cells among popliteal lymph node cells. **(C)** Release of IFN-γ from restimulated lymph node cells. Data are presented as mean±SD of triplicate cultures and are representative of 2 experiments with n = 3 mice per group. Statistically significant differences were detected with two-way analysis of variance (ANOVA). *, p<0.05; **, p<0.01.

We have previously shown that skeletal muscle injury impairs the priming of Th1-like cells in the pLN when the antigen is applied subcutaneously to the footpad that is located distal to the injured skeletal muscle tissue. This impaired Th1 priming is dependent on NK cells that are recruited to the pLN after injury [12]. We investigated whether NK cells were additionally involved in the increased Th1-like priming that we observed in response to the injection of antigen into the injured skeletal muscle. Using an anti–asialo ganglioside-monosialic acid (αGM-1) antiserum, we depleted NK cells before injury or sham treatment. Normal rabbit serum was used as a control. OVA was injected into the gastrocnemius muscles 7 d after injury or sham treatment according to the experimental design (depicted in Fig 6A). The extent of NK cell depletion is shown in Fig 6B. The depletion of NK cells from mice in both groups had no effect on either the percentage (see S6A Fig) or the proliferation (see S6B Fig) of OVA-specific Th cells. As expected, an increase in the production of IFN-γ was observed in pLN cells from injured mice after restimulation with pOVA (Fig 6C). Previous depletion of NK cells had no significant effect on the release of IFN-γ from restimulated pLN cells of either group (Fig 6C). Thus, NK cells do not contribute to the increased Th1-like priming that occurs after antigen uptake in the injured muscle.

## Discussion

In the present study, we investigated the impact of local sterile inflammation, induced by mechanical skeletal muscle injury, on antigen-specific Th cell priming in proximal lymphoid organs. As is true for the inflammatory process involved in toxin-induced skeletal muscle damage [3], we observed the sequential infiltration of neutrophilic granulocytes and monocytes/macrophages into the injured tissue. Moreover, from day 4 after injury, CD11c^+^ DC-like cells appeared in the injured muscle tissue. On day 7 after injury, 10% of the CD11c^+^MHC class II^+^ DC-like cells were CD11b^-^ and might represent classical DCs, the remaining CD11b^+^ DCs partly expressed the monocytic marker Ly6C. Under inflammatory conditions, infiltrating monocytes may differentiate into DCs [15]. During this differentiation process monocytes become positive for CD11c and MHC class II molecules and reduce the expression of Ly6C [16,17]. Alternatively, monocytes may differentiate to macrophages that express similar surface markers as reported for monocyte-derived DCs. Therefore, the nomenclature of these monocyte-derived cells is under debate [18]. We observed a population of Ly6C^+^CD11b^+^ cells in the injured muscle that heterogeneously expressed CD11c and MHC class II. We suggest that this inhomogeneous population of monocyte-derived cells contained monocyte-derived DCs and macrophages. In line with our mechanically induced injury, the appearance of monocyte-derived cells in the skeletal muscle has also reported for other models of sterile inflammation such as toxin-induced injury [19] and i.m. application of adjuvant for vaccination [20].

Although we know that DC-like cells are present in regenerating skeletal muscle tissue, their function is largely unknown. We show here for the first time that the DC-like cells that appear in the injured skeletal muscle mature *in vivo* as they exhibit a time-dependent increase in the expression of the costimulatory molecules CD40 and CD86. On day 7 after injury, mature DC-like cells were almost completely found in the CD11b^+^ subpopulation. In contrast, the CD11c^-^CD11b^+^ monocyte/macrophage population only weakly expressed CD40 and CD86. TLR agonists, such as pathogen-associated molecular patterns (PAMPs) and endogenous damage-associated molecular patterns (DAMPs), are well-known inducers of DC maturation [21,22], which is additionally driven by the presence of IFN-γ [23–26]. Because the drop weight in our injury model did not damage the natural barrier of the skin, it is unlikely that PAMPs from invading pathogens were responsible for the maturation of the accumulating DCs in the skeletal muscle.

We have previously shown that heat shock proteins (HSP) 60 and HSP70 are released from injured skeletal muscle tissue of patients undergoing orthopedic surgery [27]. It is likely that HSPs and other DAMPs were also released in our murine model of skeletal muscle injury. TLR4 is the target of several DAMPs and may contribute to sterile inflammation and remote organ damage, e.g., after bone fracture [28]. IFN-γ is known to be produced in injured skeletal muscle tissue during regeneration [29]. DCs that appeared in the injured skeletal muscle of TLR4^-/-^ and IFN-γ^-/-^ mice expressed CD40 and CD86 to a similar extent as did DCs in wildtype mice. Therefore, both TLR4 and IFN-γ appear to be dispensable for the maturation of the DCs in the injured skeletal muscle. There exist numerous DAMPs that induce the maturation of DCs independent of TLR4 such as nucleic acids, ATP, uric acid, extracellular matrix, and mitochondria [30]. So far, it is unclear to which extent these DAMPs were present in the injured skeletal muscle and if they contributed to the maturation of DCs in the damaged tissue.

A hallmark of the function of DCs is their ability to migrate from peripheral tissues to the draining lymphoid organ. After an infectious insult, monocyte-derived DCs accumulate at the site of infection and transport pathogen-derived antigens into the draining lymph node, where they induce protective immunity [16]. Considering the appearance of DC-like cells in the injured skeletal muscle, we asked whether these cells likewise migrated to the draining lymph node. Using a toxin-induced injury model, Brigitte et al. recently demonstrated that DC-like cells isolated from the injured muscle migrate into the lymph node after adoptive transfer into recipient mice [19]. Liao et al. observed a higher number of DCs in the draining lymph nodes after muscle crush injury, a model that consists of a skin incision followed by the destruction of the entire muscle [31]. On the basis of this indirect evidence, the authors assumed that DCs spontaneously migrate from the injured muscle into the draining lymph node [31].

The above mentioned results from Brigitte et al. and Liao et al. are in contrast to our previous finding that mechanically induced skeletal muscle injury, as presented here, does not change the number of DCs in the draining pLN [12]. Considering the diversity of the above-mentioned experimental approaches, we suggest that the DC-like cells in the injured skeletal muscle require a certain degree of environmental stress, such as *ex vivo* manipulation (occurring during the isolation of DCs for adoptive transfer) or a break in the natural skin barrier (allowing the invasion of pathogens) to acquire migratory ability. This assumption is supported by our novel finding that infection-induced stress, represented by injecting OVA together with the bacterial cell wall component LPS into the injured skeletal muscle, leads to an augmented accumulation of OVA-loaded DCs in the draining pLN. OVA-loaded DCs were also found in the pLN from sham-treated mice after i.m. application of the antigen, as known from vaccination studies [32], but to a significantly reduced extent. Most OVA-loaded DCs appeared in the pLN between 24 h and 48 h after injection and, thus, could be distinguished from resident DCs in the pLN, which may take up soluble antigens from lymph within minutes [33].

Therefore, we suggest that the DC-like cells that appear in the damaged tissue remain quiescent but are activated upon interaction with Gram-negative bacteria and subsequently transport pathogen-associated antigens to the draining lymph node. Further studies are required to evaluate whether the migration of these monocyte-derived cells from the injured muscle into the draining lymph node similarly takes place upon challenge with other PAMPs e.g. from Gram-positive bacteria. The retention of DC-like cells in the injured tissue under sterile conditions may protect the host against the development of autoimmunity, which would occur after spontaneous migration of mature DC-like cells into the lymph node. Moreover, when pathogens invade at the site of injury, these DC-like cells may rapidly initiate immune defense mechanisms. In this regard, we emphasize the striking difference between injury and microbial stimulation: both insults induce the accumulation of monocyte-derived DCs, but the induction of migration requires an additional signal for the DC-like cells in the injured tissue but not at the site of infection [16].

As expected, the application of OVA into the muscle of sham and injured mice stimulated OVA-specific Th cells in the pLN that subsequently proliferated and expressed the early activation marker CD69. IL-2 is released by activated Th cells prior to differentiation into a specific Th cell subset [34]. The prominent secretion of IL-2 upon restimulation with pOVA *in vitro* indicates that the differentiation of OVA-specific Th cells in the pLN of both groups was not yet completed. In parallel, the majority of OVA-specific Th cells from sham and injured mice expressed IFN-γ indicating an ongoing polarization toward Th1. Application of OVA on day 7 after injury further amplified the OVA-specific Th1-like response and the expression of the late activation marker CD25 on the OVA-specific Th cells possibly through the increased number of CD11b^+^ DC that had migrated from the injured muscle into the pLN. This is in line with a recent study from Langlet et al. who demonstrated the relevance of migratory monocyte-derived DCs in the stimulation of IFN-γ-producing T cells in a model of i.m. vaccination [20]. The Th1-like cells that are primed in the draining lymph node by DC-like cells immigrating from the injured skeletal muscle upon an infectious insult might subsequently home to the site of infection where they support pathogen clearance through increased release of IFN-γ [35].

In parallel to the enhanced OVA-specific production of IFN-γ, we observed an enlarged release of IL-10. In line with the comparable release of IL-2, OVA-specific Th cells proliferated to the same extent in injured and sham-treated mice, a finding indicating that the enhanced cytokine release was not mediated by an increase in the number of specific Th cells in the pLN. Because IL-10 is known to dampen the production of IFN-γ and other inflammatory cytokines [36], the simultaneous increase of IFN-γ and IL-10 after i.m. injection of the antigen appears to be contradictory. Recently, Th1 cells that secrete IFN-γ and IL-10 have been reported to develop during T cell priming in the presence of high antigen concentrations [37]. Increased priming of such IFN-γ^+^IL-10^+^ Th1 cells after the injection of OVA into the injured muscle may have led to the simultaneous release of both cytokines. Further studies are necessary to prove the existence of such double-positive Th1 cells in our injury model.

Adoptively transferred antigen-loaded BMDCs from naive mice migrated into the pLN of both injured and sham-treated mice to a similar extent but induced stronger Th1-like priming in the pLN when they were injected into the injured muscle. This finding indicates that the microenvironment in the injured muscle plays an important role: it seems to predispose DCs to an enhanced ability to stimulate Th cells. To date, we have no evidence of the nature of the factor(s) that primed the BMDCs in the injured muscle. Additional studies are necessary to determine whether the environmental signals that induce maturation of the endogenous DC-like cells in the injured muscle are identical to those that prime the BMDCs after adoptive transfer.

We have recently shown that the same model of skeletal muscle injury in combination with LPS-containing OVA as foreign antigen impairs the development of Th1 responses in the pLN when OVA is injected distal to the site of injury, i.e., into the hind footpads [12]. This suppressed Th1 priming is evident as soon as 24 h after injury and is maintained for at least 7 d [12]. When OVA was injected into the injured muscle 24 h after injury the extent of Th1-like priming in the pLN was less than in sham mice probably due to the injury-induced suppressive environment in the pLN at this early time point [12]. The opposite finding was observed on day 7 after injury when the i.m. application of OVA induced an increased Th1-like response indicating that the injury-induced suppression of Th1-like priming in the pLN was overcome. As mentioned above, the increased Th1-like priming on day 7 after injury was associated with the migration of antigen-loaded DC-like cells from the skeletal muscle into the pLN whereas such cells were absent in the muscle 24 h after injury. Resident DCs in the lymph nodes rapidly take up soluble antigens from lymph or blood and present them to T cells but are not sufficient to induce robust Th cell priming unless they cooperate with migratory antigen-loaded DCs that are recruited from the periphery to the lymph node [38]. Thus, differences in the functions of resident and migratory DCs may account for the diverse Th cell responses after injury depending on the site of antigen uptake (skeletal muscle or footpad).

In this regard, we found no evidence for the modulation of the ability of resident APCs to stimulate Th cells after injury *in vivo*, as observed after i.v. application of the antigen, or *in vitro* [12]. Referring to the function of migratory DCs, we must distinguish the origin of the respective DC populations. We have recently shown that the Th1 priming in the pLN that is induced by migratory cutaneous DCs (after footpad injection of the antigen) is impaired by NK cells that accumulate in the pLN after injury [12]. In contrast, the increased Th1-like priming in the pLN that was mediated by migratory DCs derived from the regenerating skeletal muscle was not affected by NK cells. Therefore, we propose that the type of migratory DCs and the participation of NK cells are decisive for the direction of the developing Th cell response in the pLN after injury.

In summary, DC-like cells that accumulate in the injured tissue during regeneration undergo maturation and, upon targeting with a foreign antigen in the presence of LPS, migrate into the draining lymph node, where they promote antigen-specific Th1-like priming. Consequently, these DC-like cells may enable the host to overcome the systemic immunosuppression induced by injury, which is a relevant complication associated with severe injury in humans [39].

## Supporting Information

S1 Fig Characterization of leukocytes in the injured muscle. (TIF)

S2 Fig The maturation of dendritic cells in the muscle after injury is independent of Toll-like receptor 4 (TLR4) and interferon (IFN) γ. (TIF)

S3 Fig Synthesis of Interferon (IFN) γ in ovalbumin-specific T helper cells and natural killer cells. (TIF)

S4 Fig T helper cell type 2-associated cytokine secretion of restimulated lymph node cells. (TIF)

S5 Fig Weak proliferation of antigen-specific T helper cells after systemic application of ovalbumin. (TIF)

S6 Fig Percentage and proliferation of antigen-specific T helper cells in the absence or presence of natural killer cells. (TIF)
