# Supplementary figures and images for: Dendritic Cell-Like Cells Accumulate in Regenerating Murine Skeletal Muscle after Injury and Boost Adaptive Immune Responses Only upon a Microbial Challenge
Source: PLoS One. 2016 May 19;11(5):e0155870. doi: 10.1371/journal.pone.0155870 (PMC4873214; doi:10.1371/journal.pone.0155870)

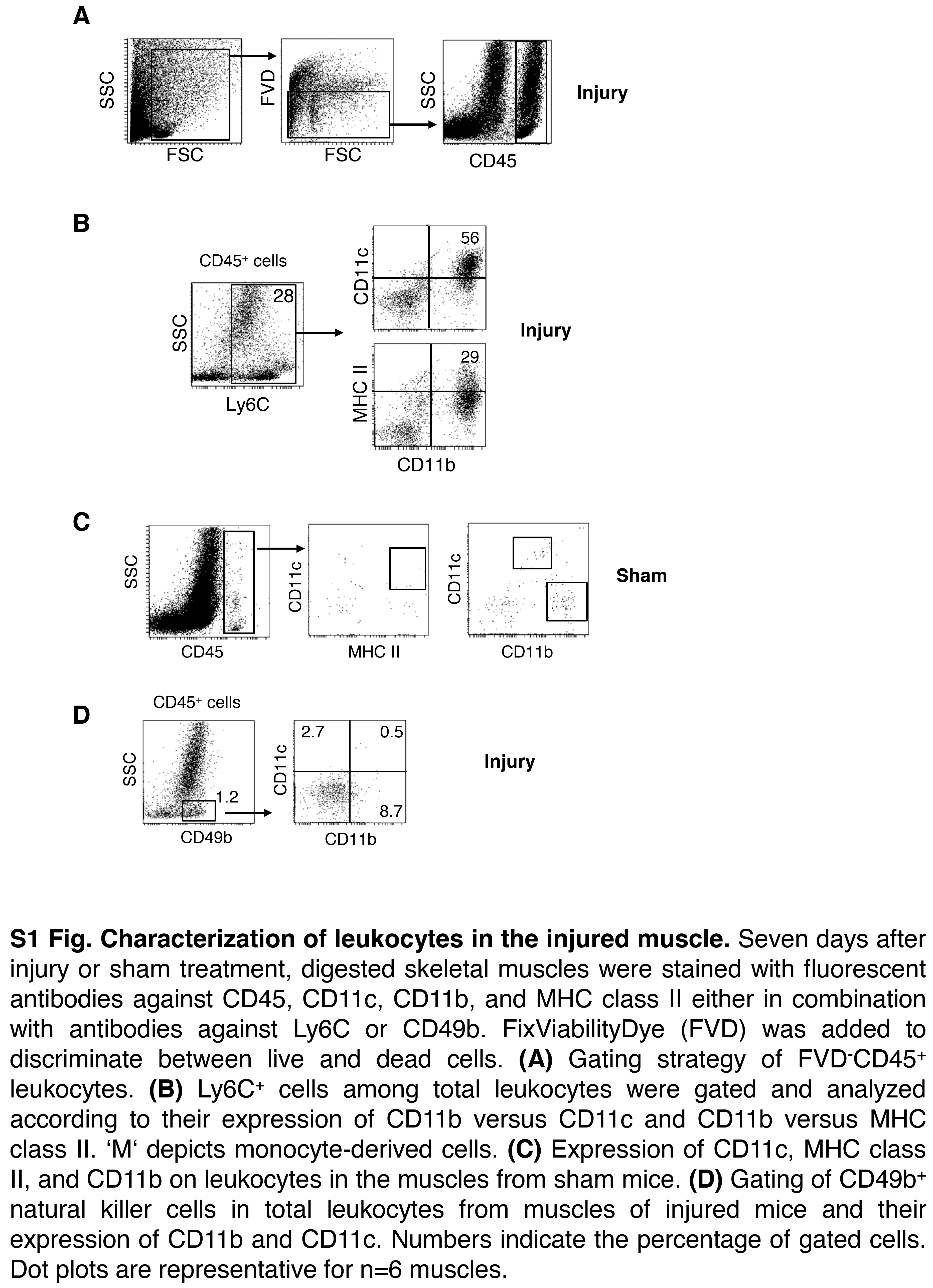

Supplement: S1 Fig — (TIF) [file pone.0155870.s001.tif]

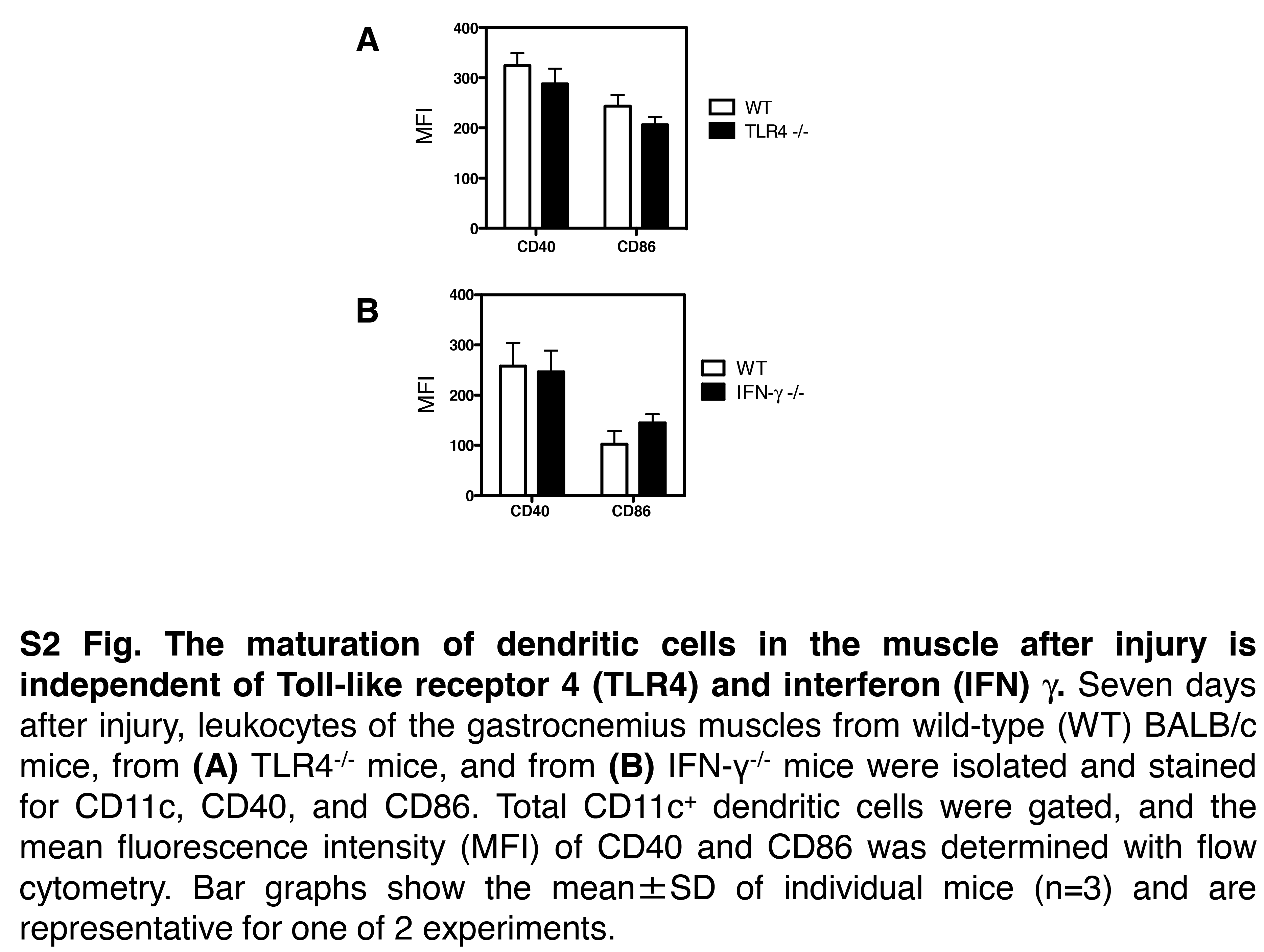

Supplement: S2 Fig — (TIF) [file pone.0155870.s002.tif]

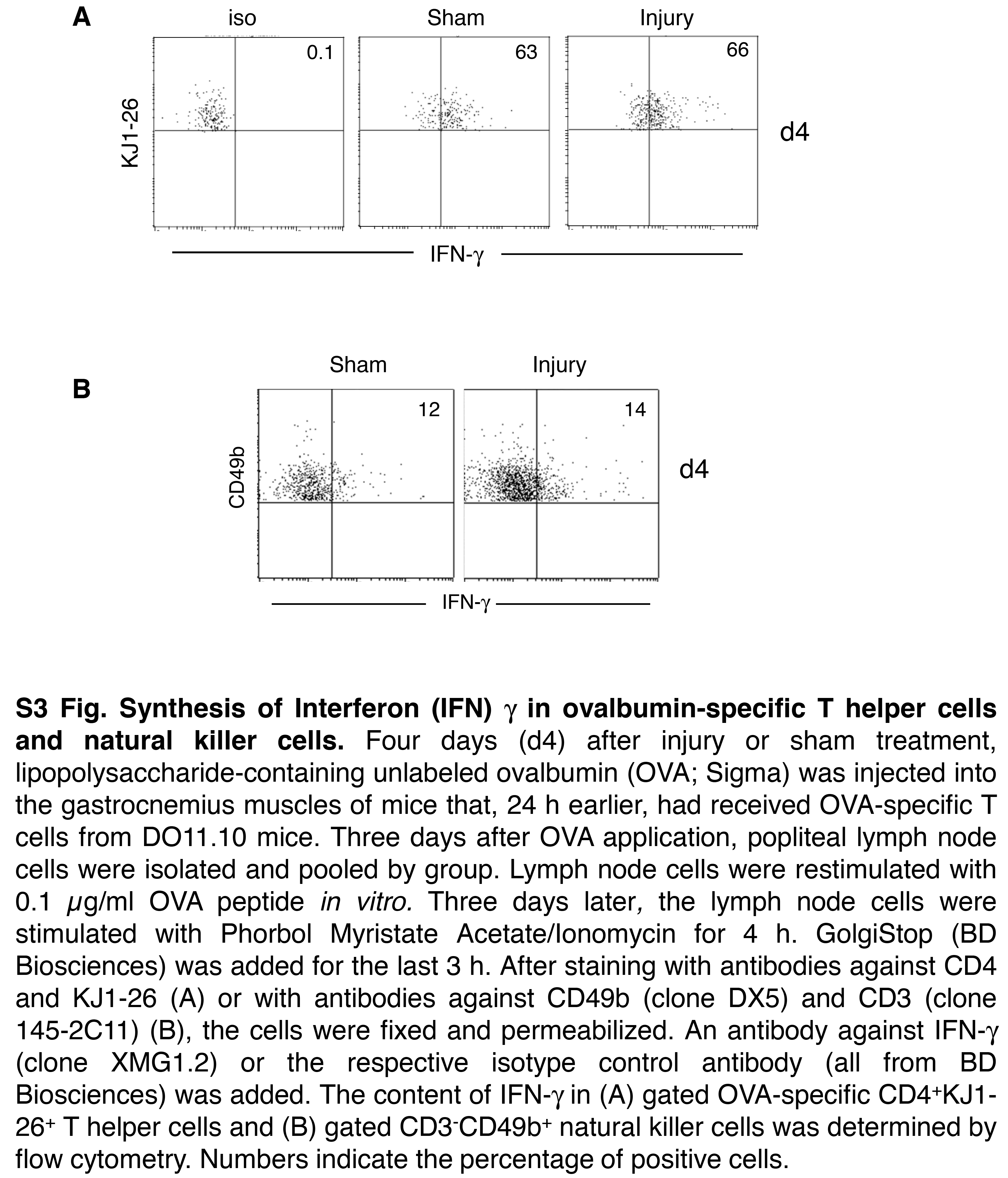

Supplement: S3 Fig — (TIF) [file pone.0155870.s003.tif]

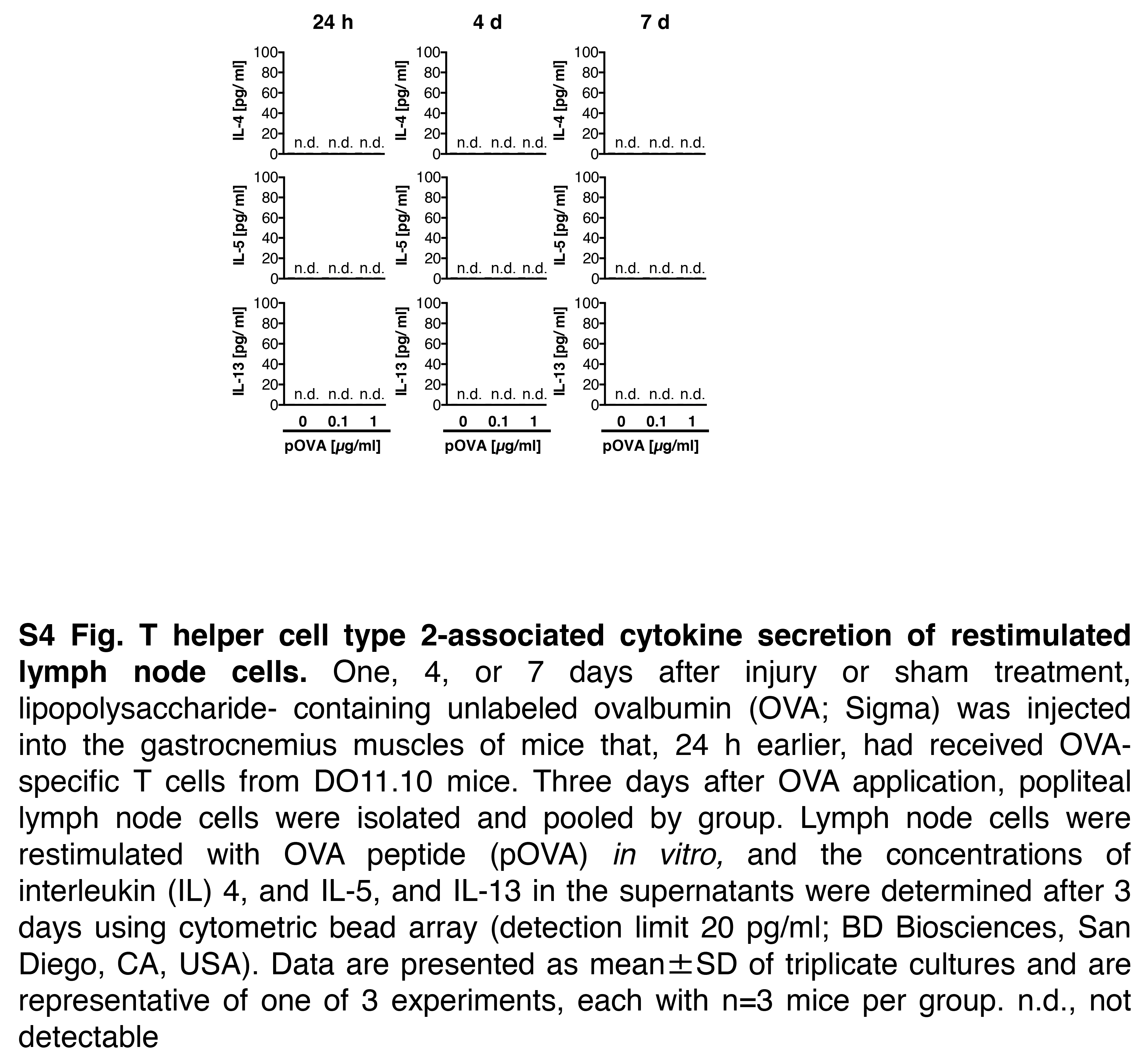

Supplement: S4 Fig — (TIF) [file pone.0155870.s004.tif]

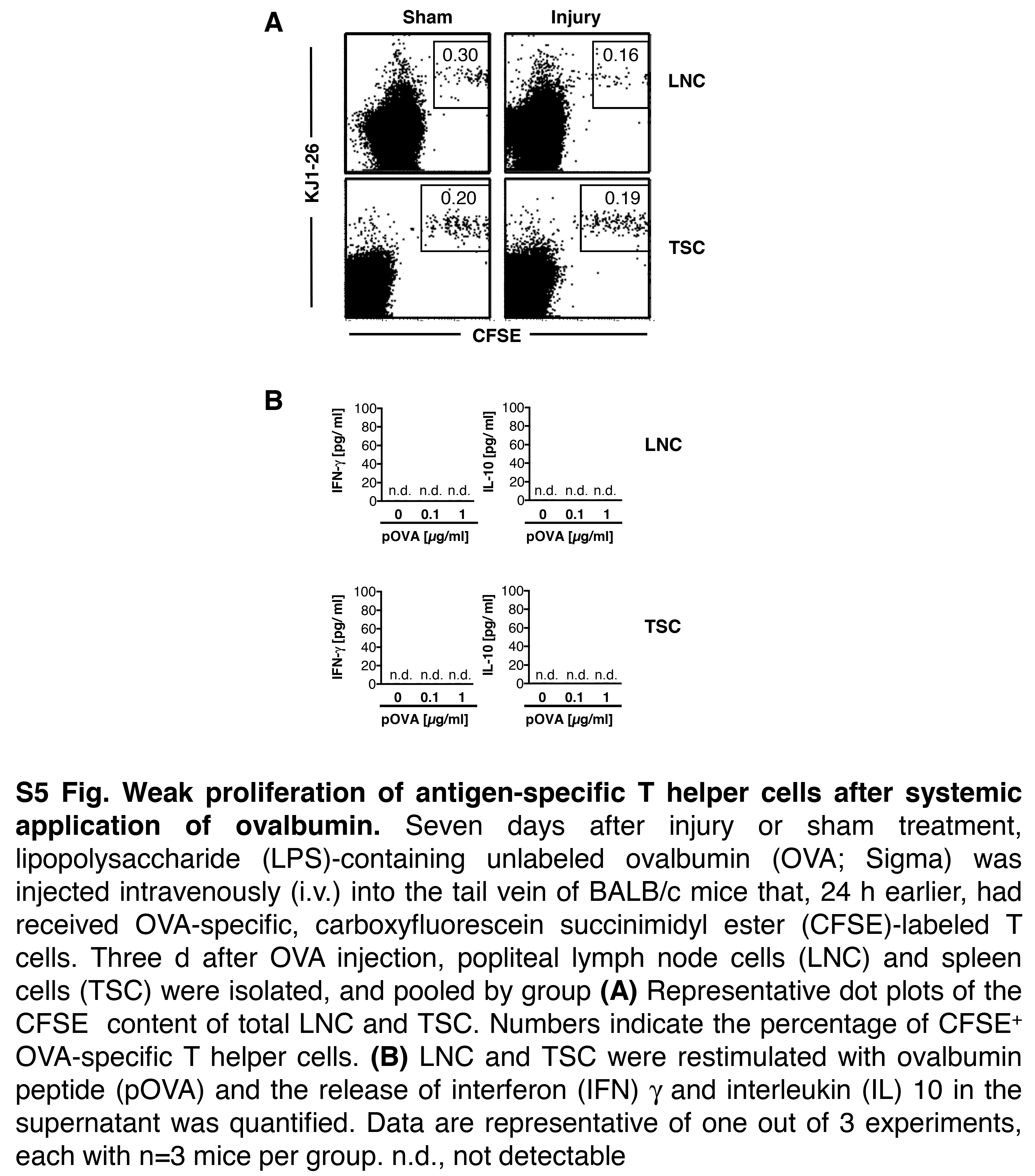

Supplement: S5 Fig — (TIF) [file pone.0155870.s005.tif]

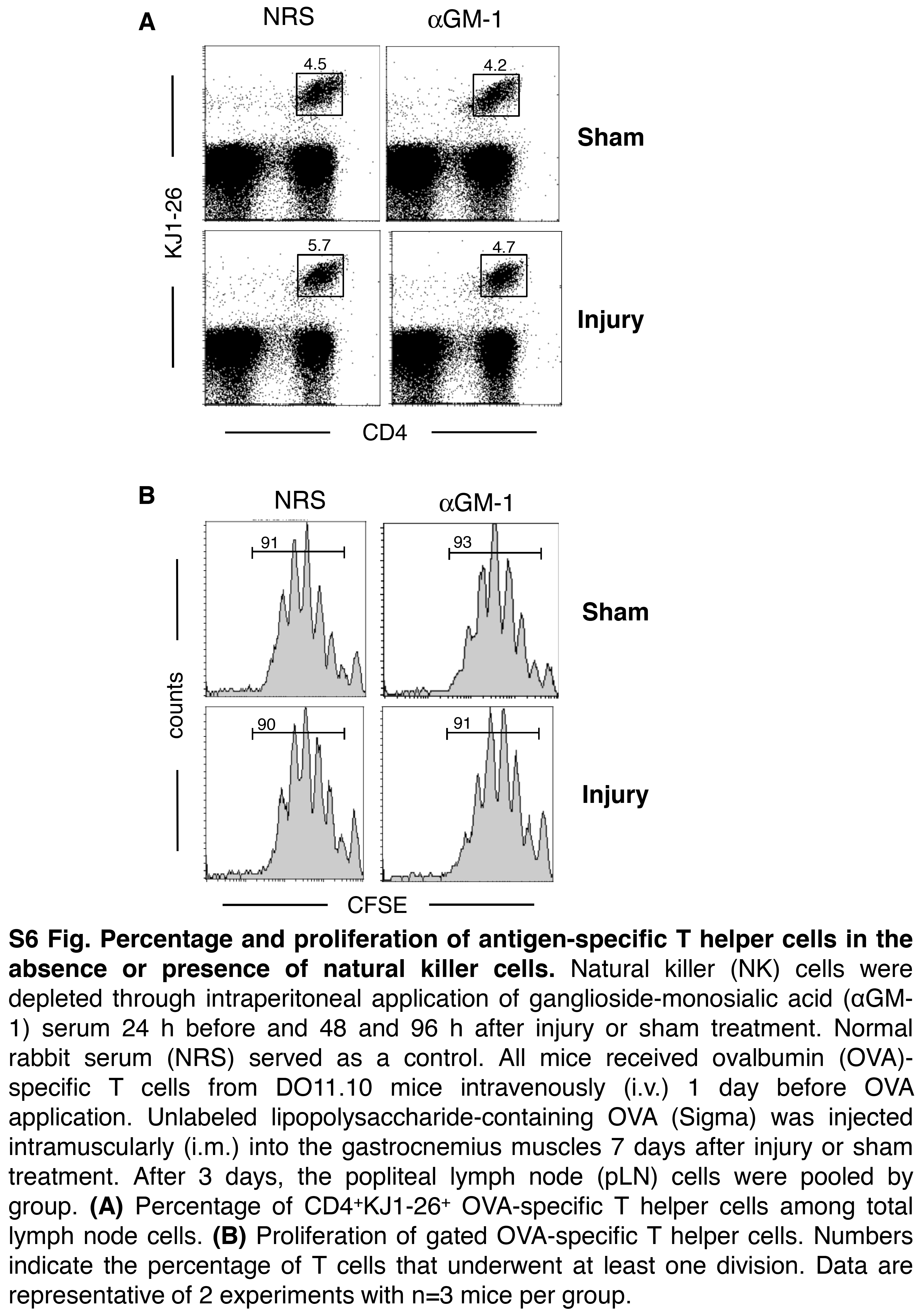

Supplement: S6 Fig — (TIF) [file pone.0155870.s006.tif]
